# Supplementary material for: Development and psychometric properties of the Japanese Consumer Assessment of Healthcare Providers and Systems Clinician & Group Survey (CG-CAHPS)
Source: PLoS One. 2021 Apr 28;16(4):e0250843. doi: 10.1371/journal.pone.0250843 (PMC8081233; doi:10.1371/journal.pone.0250843)
Supplement: S1 Appendix — (PDF) [file pone.0250843.s001.pdf]

あなたの医師について

<u>この医療機関とあなたの担当医師</u>について、調査票の質問に回答してください。

健康診断が必要なときや、健康上の問題についてアドバイスを受けたいとき、または病気やけがをしたときに、あなたは、いつもこの医師を受診しますか。

1 ☐ はい
2 ☐ いいえ

あなたは、この医師にどのくらいの期間かかっていますか。

1 ☐ 6 ヶ月未満
2 ☐ 6 ヶ月以上 1 年未満
3 ☐ 1 年以上 3 年未満
4 ☐ 3 年以上 5 年未満
5 ☐ 5 年以上

過去 6 ヶ月間の外来受診について

以下は、あなた自身の<u>外来受診</u>についての質問です。

過去 6 ヵ月間に、あなたは、この医師を何回受診しましたか。

0 ☐ 0 回 → **質問 19 へ**
1 ☐ 1 回
2 ☐ 2 回
3 ☐ 3 回
4 ☐ 4 回
5 ☐ 5～9 回
6 ☐ 10 回以上

1. 過去 6 ヵ月間に、<u>直ちに診察が必要な病気やけが</u>などのために、この医療機関の受診予約をとったことがありますか。

1 ☐ はい
2 ☐ いいえ → **質問 3 へ**

2. 過去 6 ヵ月間に、<u>直ちに診察が必要</u>で、この医療機関の受診予約をとったとき、すぐに予約を入れることができましたか。

1 ☐ 一度もそうではなかった
2 ☐ 時々はそうだった
3 ☐ たいていはそうだった
4 ☐ いつもそうだった

3. 過去 6 ヵ月間に、<u>健康診断や定期診察</u>のために、この医療機関の受診予約をとったことがありますか。

1 ☐ はい
2 ☐ いいえ → **質問 5 へ**

4. 過去 6 ヵ月間に、健康診断や定期診察で、この医療機関の受診予約をとったとき、すぐに予約を入れることができましたか。

1☐ 一度もそうではなかった
2☐ 時々はそうだった
3☐ たいていはそうだった
4☐ いつもそうだった

5. 過去 6 ヵ月間に、医学的な相談をするため、診療時間内に、この医療機関に連絡をとったことがありますか。

1☐ はい
2☐ いいえ → **質問 7 へ**

6. 過去 6 ヵ月間に、診療時間内にこの医療機関に連絡をとったとき、医学的な相談に対する回答をその日のうちにもらうことができましたか。

1☐ 一度もそうではなかった
2☐ 時々はそうだった
3☐ たいていはそうだった
4☐ いつもそうだった

7. 過去 6 ヶ月間に、この医師は、あなたにわかりやすく説明をしましたか。

1☐ 一度もそうではなかった
2☐ 時々はそうだった
3☐ たいていはそうだった
4☐ いつもそうだった

8. 過去 6 ヶ月間に、この医師は、あなたの話を注意深く聴きましたか。

1☐ 一度もそうではなかった
2☐ 時々はそうだった
3☐ たいていはそうだった
4☐ いつもそうだった

9. 過去 6 ヶ月間に、この医師は、あなたの病歴についての重要な情報を知っているようでしたか。

1☐ 一度もそうではなかった
2☐ 時々はそうだった
3☐ たいていはそうだった
4☐ いつもそうだった

10. 過去 6 ヶ月間に、この医師は、あなたの話に対して敬意を示しましたか。

1 一度もそうではなかった
2 時々はそうだった
3 たいていはそうだった
4 いつもそうだった

11. 過去 6 ヶ月間に、この医師は、あなたのために時間を十分にとりましたか。

1 一度もそうではなかった
2 時々はそうだった
3 たいていはそうだった
4 いつもそうだった

12. 過去 6 ヵ月間に、この医師は、あなたに対して血液検査やレントゲンなどの検査を実施しましたか。

1 はい
2 いいえ → **質問 14 へ**

13. 過去 6 ヵ月間に、この医師が、あなたに対して血液検査やレントゲンなどの検査を実施した際に、この医療機関の職員は、検査結果の説明の手助けをしましたか。

1 一度もそうではなかった
2 時々はそうだった
3 たいていはそうだった
4 いつもそうだった

14. この医師を 0 から 10 点で評価してください（最も悪い場合を 0 点、最も良い場合を 10 点とします）。

0 0 最も悪い医師
1 1
2 2
3 3
4 4
5 5
6 6
7 7
8 8
9 9
10 10 最も良い医師

15. 過去 6 ヵ月間に、あなたには薬が処方されましたか。

1 はい
2 いいえ → **質問 17 へ**

16. 過去 6 ヵ月間に、あなたが服用するすべての処方薬について、この医療機関の職員と話をしましたか。

1 一度もそうではなかった
2 時々はそうだった
3 たいていはそうだった
4 いつもそうだった

医療機関の事務職員について

17. 過去 6 ヶ月間に、この医療機関の事務職員は、あなたの期待通りの手助けをしましたか。

1☐ 一度もそうではなかった
2☐ 時々はそうだった
3☐ たいていはそうだった
4☐ いつもそうだった

18. 過去 6 ヶ月間に、この医療機関の事務職員は、礼儀と敬意をもって、あなたに接しましたか。

1☐ 一度もそうではなかった
2☐ 時々はそうだった
3☐ たいていはそうだった
4☐ いつもそうだった

あなたについて

19. 全体的にみて、あなたの健康状態はどうですか。

1☐ 最高に良い
2☐ とても良い
3☐ 良い
4☐ ふつう
5☐ 悪い

20. 全体的にみて、あなたの精神的な健康状態はどうですか。

1☐ 最高に良い
2☐ とても良い
3☐ 良い
4☐ ふつう
5☐ 悪い

21. あなたの年齢を答えてください。

1☐ 18～24 歳
2☐ 25～34 歳
3☐ 35～44 歳
4☐ 45～54 歳
5☐ 55～64 歳
6☐ 65～74 歳
7☐ 75 歳以上

22. あなたの性別を答えてください。

1☐ 男性
2☐ 女性

23. あなたの最終学歴を答えてください。

1☐ 小学校・中学校
2☐ 高等学校
3☐ 専門学校または短期大学
4☐ 4 年制大学以上

これで調査は終わりです。
ご協力ありがとうございました。
